# Association between Intake of Sugar-Sweetened Beverages and Circulating 25-Hydroxyvitamin D Concentration among Premenopausal Women

**DOI:** 10.3390/nu6082987

**Published:** 2014-07-28

**Authors:** Caroline S. Duchaine, Caroline Diorio

**Affiliations:** 1 Cancer Research Center, Department of Social and Preventive Medicine, Laval University, Quebec City, Quebec G1V 0A6, Canada; E-Mail: caroline.duchaine.1@ulaval.ca; 2 Oncology Unit, CHU de Québec Research Center, Saint-Sacrement Hospital, Quebec, QC G1S 4L8, Canada; 3 Deschênes-Fabia Center for Breast Diseases, Saint-Sacrement Hospital, Quebec, QC G1S 4L8, Canada

**Keywords:** 25-hydroxyvitamin D, vitamin D, carbonated beverages, fructose, premenopausal period, dietary sugars

## Abstract

Intake of sugar-sweetened beverages has increased in North America and seems to have several adverse health effects possibly through decreased circulating 25-hydroxyvitamin D (25(OH)D) concentrations. The aim of this cross-sectional study was to evaluate the association between sugar-sweetened beverages intake and 25(OH)D concentrations among premenopausal women. Intake of sugar-sweetened beverages including colas, other carbonated beverages and sweet fruit drinks was assessed using a validated food frequency questionnaire among 741 premenopausal women. Plasma concentrations of 25(OH)D were quantified by radioimmunoassay. The association between sugar-sweetened beverages intake and 25(OH)D concentrations was evaluated using multivariate generalized linear models and Spearman correlations. A higher intake of colas was associated with lower mean 25(OH)D levels (67.0, 63.7, 64.7 and 58.5 nmol/L for never, <1, 1–3 and >3 servings/week, respectively; *r* = −0.11 (*p* = 0.004)). A correlation was observed between intake of other carbonated beverages and 25(OH)D concentrations but was not statistically significant (*r* = −0.06 (*p* = 0.10)). No association was observed between intake of sweet fruit drinks and 25(OH)D concentrations. This study suggests that high intake of colas may decrease 25(OH)D levels in premenopausal women. Considering the high consumption of these drinks in the general population and the possible consequences of vitamin D deficiency on health, this finding needs further investigation.

## 1. Introduction

Consumption of sugar-sweetened beverages has increased considerably in the past 30 years and has become one of the main types of beverages in North America [1]. In the United States, a citizen consumes on average 12 servings of soda per week of which 58% contain sugar [2]. In Canada, soft drinks have been the leading beverage choice between 1980 and 2000 [1] and they remain so today [3]. This excessive intake of sugar-sweetened beverages may have several adverse effects on human health such as diabetes, hypertension [2], obesity, cardiovascular diseases [4], low bone mineral density [5], hypocalcaemia [6,7] and some types of cancer [8,9,10,11].

Interestingly, the link between insufficient circulating 25-hydroxyvitamin D (25(OH)D) concentrations and similar health outcomes was established with low bone mineral density [12], and proposed with diabetes [13], metabolic syndrome [14], hypertension [15] and cancer [16,17,18,19,20,21]. The 25(OH)D is the principal circulating vitamin D metabolite and a well-established biomarker for total vitamin D exposure from food, supplements and endogenous synthesis [22]. The Institute of Medicine report on dietary reference intake for calcium and vitamin D recommends 25(OH)D concentrations of 50 nmol/L or more for good health and establishes the deficiency limit at 30 nmol/L [12]. In Canada, 25.7% of the population had plasma concentrations of 25(OH)D below 50 nmol/L in 2011 [23]. Thus, an inter-individual fluctuation in 25(OH)D concentrations may modulate the risk of some diseases, and this fluctuation could be due to genetic or environmental/lifestyle factors including nutrition [22].

In fact, some studies found that intake of sugar-sweetened beverages could have an impact on circulating concentrations of 25(OH)D. Sugar-sweetened beverages, particularly colas, have been shown to decrease concentrations of plasma 25(OH)D in rats [24]. Following a two-month diet, rats consuming cola beverages had significantly lower plasma 25(OH)D concentrations than rats consuming water (*p* < 0.001). Furthermore, one cross-sectional study among children has shown an inverse association of intake of soda (*p* < 0.001) and juice (*p* = 0.009) with serum concentration of 25(OH)D [25]. Some of the nutrients contained in sugar-sweetened beverages, like fructose, caffeine and phosphoric acid, have already been proposed to explain the link of such beverages with circulating concentrations of 25(OH)D [24,26,27,28]. To further explore this link, we have evaluated the association of intake of sugar-sweetened beverages (colas, other carbonated beverages and sweet fruit drinks separately or combined) with plasma concentrations of 25(OH)D in a population of 741 premenopausal women.

## 2. Experimental Section

### 2.1. Study Population and Recruitment Procedure

Study population and recruitment procedure have been described elsewhere [29,30]. Briefly, premenopausal women were recruited between February and December 2001 at a private radiology clinic among those who received a screening mammogram. Exclusion criteria were personal history of cancer or breast surgery, having used hormonal derivatives within three months prior to mammography, having used tamoxifen or raloxifene, being pregnant, having diabetes mellitus, dwarfism/acromegaly, thyroid, adrenal, or hepatic disease. Among the 777 premenopausal women recruited, 741 provided a written informed consent to use their blood sample for assays other than those planned at recruitment. This study was approved by the Research Ethics Review Board-Hôpital du Saint-Sacrement du CHU de Québec.

### 2.2. Data Collection

Blood sample, height, weight, and waist and hip circumferences were collected at recruitment by a trained nurse. Information on smoking status, alcohol intake and education were collected during a phone interview performed by trained interviewers. Physical activity was assessed using the Nurses’ Health Study II Activity and Inactivity Questionnaire [31] and then expressed as metabolic equivalent hours per week (MET-h/week) [32].

### 2.3. Diet Assessment

Information on diet was documented with a 161 item validated [33,34], self-administered semi-quantitative food frequency questionnaire (97GP copyrighted at Harvard University, Boston, MA, USA). Nutrient content of food was assessed at Harvard University, using U.S. Department of Agriculture sources, supplemented with data from food manufacturers and personal communications with laboratories. A standard serving size was specified for each item and its frequency of consumption over the previous year, ranging from “never” to “more than three servings per day”, was reported. From this food frequency questionnaire, four items were classified as sugar-sweetened beverages: colas with sugar containing caffeine, colas with sugar but caffeine-free, carbonated beverages with sugar other than colas and sweet fruit drinks.

### 2.4. Assessment of Plasma 25(OH)D

At the time of collection, samples of blood were rapidly aliquoted and stored at −80°C for subsequent analyses. Plasma 25(OH)D concentrations (nmol/L) were quantified by radioimmunoassay after acetonitrile extraction (DiaSorin Inc, Stillwater, MN, USA) between November 2005 and January 2006. The results met the performance target set by the International 25-Hydroxyvitamin D External Quality Assessment Scheme Advisory Panel in 2004–2005. Four blinded duplicates on average were made for each of the 24 batches and the intra-batch and between-batch coefficients of variation were 7.3% and 8.8%, respectively.

### 2.5. Statistical Analyses

The frequency of consumption of each sugar-sweetened beverage was transformed in number of servings per week using the middle of each category when necessary. For example, when the response category was 1–3 servings per week, we assigned two servings per week to this data. The number of servings per week of each item was categorized as never, <1, 1–3 and >3 servings/week or to ensure at least 5% of the population in each group. Since very few women consumed one serving of caffeine-free cola or more per week (*n* = 11), this item was combined with regular cola intake. The sum of servings per week of all sugar-sweetened beverages was also calculated and categorized according to the same criteria. Crude and adjusted mean concentrations of 25(OH)D for each category of servings per week were estimated using generalized linear models, and the *p*-value for each estimate compared to the reference category is provided. Spearman coefficients were used to assess the correlation between intakes of each sugar-sweetened beverage item and 25(OH)D concentrations as continuous variables. Determinants of 25(OH)D concentrations identified *a priori* [35,36,37] and included in the adjusted models were age (year) at recruitment, body mass index (BMI) (kg/m^2^), waist-to-hip ratio, total vitamin D (UI/day) and total calcium (mg/day) intakes from food and supplements in the past year, total caloric (kcal/day) intake in the past year, season at blood sample collection and leisure-time physical activity (MET-h/week) in the past year (as proxy variables of sun exposure), alcohol intake (servings/week) in the past year, education (highest completed degree: primary, secondary, college, university), and smoking status (non, former or current smoker). In order to account for the possible displacement of milk in the diet by sugar-sweetened beverages, Spearman correlations further adjusted for milk intake were also estimated. Women with missing values were excluded from analyses. All tests were 2-sided and a *p* < 0.05 was considered statistically significant. All statistical analyses were carried out using SAS 9.3 (SAS Institute, Inc., Cary, NC, USA) software system.

## 3. Results

Characteristics of the study population are shown in Table 1. The mean age at recruitment was 46.8 years and the women presented a BMI of 25.2 kg/m^2^ on average. The mean plasma concentration of 25(OH)D was 65.0 nmol/L, 23.2% (*n* = 172) of the women had 25(OH)D concentrations below the 50 nmol/L recommended and 1.75% (*n* = 13) were below the deficiency level of 30 nmol/L. Regarding calcium intake, the mean concentration in this study population (974.6 mg/day) was below the dietary reference intake recommended by the Institute of Medicine report (1000–1200 mg/day for women aged between 30 and 70 years). Most of the women (43.2%) in this population were non-consumers of colas (regular or caffeine free) or consumed less than one serving per week of this type of beverage (36.7%). Similar patterns were observed for other carbonated beverages and sweet fruit drinks with 44.2% and 50.7% for non-consumers and 44.3% and 35.6% for less than one serving per week, respectively.

**Table 1:** Characteristics of the study population.

Premenopausal women (*n* = 741) ^1^	
Age (year), mean (SD)	46.8 (4.6)
Body mass index (kg/m^2^), mean (SD)	25.2 (4.5)
Waist-to-hip ratio, mean (SD)	0.78 (0.06)
Plasma 25(OH)D concentrations, (nmol/L), mean (SD)	65.0 (19.6)
Vitamin D intake ^2^ (IU/day), mean (SD)	284.3 (231.6)
Calcium intake ^2^ (mg/day), mean (SD)	974.6 (435.0)
Milk intake ^3^ (servings/week), mean (SD)	5.5 (5.9)
Caloric intake (kcal/day), mean (SD)	1906 (514)
Physical activity (MET-h/week), mean (SD)	27.2 (22.2)
Alcohol (servings/week), mean (SD)	3.4 (3.8)
*Total colas* *^4^ (servings/week), n (%)*	
Never	319 (43.2)
<1	271 (36.7)
1–3	103 (13.9)
>3	46 (6.2)
*Other sweet carbonated beverages (servings/week), n (%)*	
Never	327 (44.2)
<1	328 (44.3)
≥1	85 (11.5)
*Sweet fruit drinks (servings/week), n (%)*	
Never	375 (50.7)
<1	263 (35.6)
≥1	101 (13.7)
*Total of all sugar-sweetened beverages* *^5^ (servings/week), n (%)*	
Never	130 (17.7)
<1	304 (41.3)
1–3	154 (20.9)
>3	148 (20.1)
*Season at blood sampling, n (%)*	
Winter	90 (12.2)
Spring	249 (33.6)
Summer	184 (24.8)
Fall	218 (29.4)
*Education, n (%)*	
Less than a high school degree	48 (6.5)
High school degree	235 (31.7)
College degree	197 (26.6)
University degree	261 (35.2)
*Smoking status, n (%)*	
Nonsmoker	337 (45.5)
Ex-smoker	294 (39.7)
Current smoker	110 (14.8)

Abbreviations: 25(OH)D = 25-hydroxyvitamin D, IU/day = International unit per day, MET-h/week = metabolic equivalent hours per week. ^1^ Missing values for caffeine free colas (*n* = 2), other carbonated beverages (*n* = 1), sweet fruit drinks (*n* = 2). ^2^ Total intakes from food and supplements. ^3^ Including skim milk, 1% and 2% milk and full cream milk. ^4^ Including regular colas and caffeine free colas. ^5^ Including regular colas, caffeine free colas, other carbonated beverages and sweet fruit drinks.

Table 2 shows relations of intake of sugar-sweetened beverages and plasma 25(OH)D concentrations. In adjusted models, a higher intake of colas was associated with lower mean concentrations of plasma 25(OH)D (67.0, 63.7, 64.7 and 58.5 nmol/L for never, <1, 1–3 and >3 servings/week, respectively; *r* = −0.11 (*p* = 0.004)). Consumption of carbonated beverages other than colas was negatively correlated with the concentrations of 25(OH)D (*r* = −0.06), but the association was not statistically significant (*p* = 0.10). No association was observed of intake of sweet fruit drinks or total intake of sugar-sweetened beverages with plasma concentrations of 25(OH)D (*r* = −0.03 (*p* = 0.48) for fruit drinks and *r* = −0.06 (*p* = 0.09) for total of beverages). Further adjustment for milk consumption did not materially alter our results.

**Table 2:** Relations of intake of sugar-sweetened beverages with plasma 25(OH)D concentrations.

Sugar-sweetened beverages (Servings/week)	*n*	Crude	Adjusted ^1^
		25(OH)D (nmol/L) mean (95% CI), *p* ^2^	25(OH)D (nmol/L) mean (95% CI), *p* ^2^
*Total of all colas ^3^*			
Never	319	67.5 (65.3–69.6), ref.	67.0 (65.2–68.9), ref.
<1	271	63.6 (61.3–65.9), 0.02	63.7 (61.8–65.7), 0.02
1–3	103	66.0 (62.3–65.9), 0.51	64.7 (61.4–67.9), 0.21
>3	46	54.4 (48.9–60.0), <0.0001	58.5 (53.4–63.6), 0.003
Spearman *r* (*p*)		−0.13 (0.0004)	−0.11 (0.004)
Spearman *r* (*p*) ^4^			−0.11 (0.005)
*Other sweet carbonated beverages*			
Never	327	66.4 (64.3–68.5), ref.	66.1 (64.3–67.9), ref.
<1	328	64.1 (62.0–66.3), 0.14	64.1 (62.3–65.8), 0.12
≥1	85	62.6 (58.4–66.7), 0.11	63.7 (60.0–67.4), 0.26
Spearman *r* (*p*)		−0.08 (0.03)	−0.06 (0.10)
Spearman *r* (*p*) ^4^			−0.06 (0.13)
*Sweet fruit drinks*			
Never	375	65.9 (63.9–67.8), ref.	65.4 (63.6–67.0), ref.
<1	263	63.2 (60.8–65.5), 0.08	64.0 (62.0–68.8), 0.31
≥1	101	66.2 (62.4–70.0), 0.87	65.6 (62.3–68.8), 0.91
Spearman *r* (*p*)		−0.04 (0.33)	−0.03 (0.48)
Spearman *r* (*p*) ^4^			−0.03 (0.47)
*Total of all sugar-sweetened beverages ^5^*			
Never	130	66.5 (63.1–69.9), ref.	65.5 (62.6–68.4), ref.
<1	304	65.1 (62.9–67.3), 0.49	65.7 (63.8–67.6), 0.91
1–3	154	64.6 (61.5–67.7), 0.42	63.8 (61.1–66.4), 0.38
>3	148	64.0 (60.8–67.1), 0.28	64.3 (61.4–67.1), 0.56
Spearman *r* (*p*)		−0.09 (0.02)	−0.06 (0.09)
Spearman *r* (*p*) ^4^			−0.07 (0.08)

Abbreviations: 25(OH)D = 25-hydroxyvitamin D, ref. = reference group. ^1^ Adjusted for age, body mass index, waist-to-hip ratio, physical activity, alcohol intake, total vitamin D, calcium and caloric intakes, season at blood sample, smoking status and education. ^2^ *p* values obtained from generalized linear models with *t* test comparison between means of 25(OH)D concentrations for each category of sugar-sweetened beverages intake and the reference group. ^3^ Including regular colas and caffeine free colas. ^4^ Including skim milk, 1% and 2% milk and full cream milk. ^5^ Including regular colas, caffeine free colas, other carbonated beverages and sweet fruit drinks.

**Table 3:** Partial correlations between determinants of 25(OH)D concentration and plasma 25(OH)D concentrations.

Determinant of 25(OH)D concentration	Spearman correlations			
	*r* ^1^	*p* ^1^	*r* ^2^	*p* ^2^
Age (year)	−0.05	0.18	−0.05	0.17
Body mass index (kg/m^2^)	−0.14	0.0001	−0.15	<0.0001
Waist-to-hip ratio	0.02	0.66	0.02	0.63
Vitamin D intake ^3^ (IU/day)	0.13	0.0003	0.12	0.0002
Calcium intake ^3^ (mg/day)	0.15	<0.0001	0.13	0.0005
Caloric intake (kcal/day)	−0.10	0.005	−0.08	0.04
Physical activity (MET-h/week)	0.20	<0.0001	0.20	<0.0001
Alcohol (servings/week)	0.04	0.34	0.02	0.56
Season at blood sampling, *n* (%) ^4^	0.40	<0.0001	0.40	<0.0001
Education, *n* (%)	−0.12	0.001	−0.13	0.0004
Smoking status, *n* (%)	0.07	0.08	0.07	0.06
Total of all colas (serving/week) ^5^			−0.11	0.004

^1^ Adjusted for all other determinants included in this table except for total of all colas. ^2^ Adjusted for all other determinants included in this table. ^3^ Total intakes from food and supplements. ^4^ Coded winter = 1, fall and spring = 2 and summer = 3. ^5^ Including regular colas and caffeine free colas.

Spearman correlations between each determinant of 25(OH)D and plasma 25(OH)D concentration are shown in Table 3. The intake of vitamin D and calcium, season at blood sampling and physical activity were positively correlated with 25(OH)D concentrations (*r* = 0.13 (*p* = 0.0003), *r* = 0.15 (*p* = <0.0001), *r* = 0.40 (*p* = <0.0001) and *r* = 0.20 (*p* = <0.0001), respectively). Negative correlations with 25(OH)D concentrations were observed for BMI, caloric intake and education (*r* = −0.14 (*p* = 0.0001), *r* = −0.10 (*p* = 0.005) and *r* = −0.12 (*p* = 0.001), respectively). In our data, no correlation was observed between age, waist-to-hip ratio, smoking status or intake of alcohol and 25(OH)D concentrations. Adding total intake of all colas in the model slightly changed the observed correlation between education, calcium or caloric intake with 25(OH)D concentrations (*r* = −0.13 (*p* = 0.0004), *r* = 0.13 (*p* = 0.0005), *r* = −0.08 (*p* = 0.04), respectively), but all of the other correlations remained essentially unchanged.

## 4. Discussion

In this study, we found that higher intake of colas was associated with lower plasma concentrations of 25(OH)D among premenopausal women. Compared to non-consumers, women who drank more than three servings per week of colas presented a mean concentration of 25(OH)D that was 12.7% lower. In the latter category of colas consumption, 47.8% of women presented 25(OH)D concentrations below the recommended 50 nmol/L. Furthermore, 10.9% of these women had 25(OH)D concentrations below 30 nmol/L, which are considered as deficient concentrations. In comparison, only 16.3% of the women who never drank colas presented concentrations of 25(OH)D below 50 nmol/L of which only 0.3% (*n* = 1) were below 30 nmol/L.

Few studies have evaluated the association between intake of sugar-sweetened beverages and concentrations of 25(OH)D [24,25]. Following a two month diet, Garcia-Contreras and colleagues observed lower plasma 25(OH)D concentrations among rats consuming colas beverages compared to those who drank water [24]. In a cross-sectional study conducted among 411 obese children, Olson and colleagues found that both intake of sodas and juices were inversely associated with serum concentrations of 25(OH)D [25]. Our observations appear consistent with these studies conducted in population other than premenopausal women. One study, among women, has evaluated short-term effects of carbonated beverages consumption on serum concentrations of 1,25-dihydroxyvitamin D (1,25(OH)_2_D), the active form of vitamin D metabolized by the kidney and target tissues (such as breast, prostate, intestine, skin and bones) from 25(OH)D [38]. Concentrations of 1,25(OH)_2_D were lower in the group following a diet high in colas intake (1.4 L/day) compared to women following a diet without cola (85.4 nmol/L and 99.8 nmol/L, respectively), but the association was not significant. However, the study population was very small (eight women) and the serum concentrations of 1,25(OH)_2_D may not be the best biomarker to represent vitamin D concentrations [22]. Some authors have brought the idea that the effect of intake of colas on 25(OH)D concentrations was due to a replacement of the milk consumption by colas [25]. However, further adjustment for milk intake did not materially alter our results, suggesting that this factor does not confound the observed associations with 25(OH)D concentrations.

In our study, we found an inverse association of intake of colas with 25(OH)D concentrations and also a non-significant negative correlation between intake of other carbonated beverages and 25(OH)D concentrations. However, the intake of sweet fruit drinks was not associated with 25(OH)D concentrations. In comparison to sweet fruit drinks, carbonated beverages contain a higher concentration of fructose which is derived from corn syrup and used as a sweetener [39]. It has been suggested that fructose may have an effect on vitamin D metabolism [26]. In fact, the liver and kidneys, the principal sites of vitamin D metabolism, seem adversely affected by chronic fructose intake [40,41]. Moreover, one study conducted among rats with compromised renal function has shown that those fed with fructose had a reduction of 30%–40% in their 25(OH)D serum concentrations compared to rats fed with glucose [26]. This observation could explain the observed lower 25(OH)D concentrations with higher intake of carbonated beverages in our population. Furthermore, we observed no association between intake of diet colas and 25(OH)D concentrations (data not shown). However, the statistically significant association found with intake of colas was stronger than that of intake of other sweet carbonated beverages and thus may not be solely due to fructose.

A difference between colas and other sugar-sweetened beverages is that they contain caffeine which has been shown to increase the risk of osteoporosis [42,43]. To take this into consideration, we have made separate analyses of intakes of regular colas and caffeine free colas with 25(OH)D concentrations, and found similar negative correlations for both types of colas (*r* = −0.094, *p* = 0.012 for regular colas and *r* = −0.103, *p* = 0.006 for caffeine free colas). Thus, in the present study, caffeine does not seem to explain the results.

In contrast to other sugar-sweetened beverages, colas also contain phosphoric acid [5] and excessive exogenous phosphate intake is believed to cause vitamin D and calcium metabolism disorders [27,28]. One study has shown that intake of soft drinks containing phosphoric acid induced hyperphosphaturia and significant reductions in plasma concentrations of 1,25(OH)_2_D and 25(OH)D in immature rats [44]. All of these hypotheses need to be confirmed with additional studies, especially among humans.

This study has several strengths. The study sample was relatively large and the women were recruited in a short period of time. The measurement of plasma 25(OH)D concentrations was done with respect to high quality standards and the food frequency questionnaire used to assess intake of sugar-sweetened beverages was validated in other studies for its accuracy [33,34]. Several possible confounding factors (including vitamin D intake from food and supplements and milk consumption) of the association between intake of sugar-sweetened beverages and 25(OH)D concentrations were taken into account. Furthermore, the women of this study lived in the same geographic area, thus were exposed to the same climatic conditions and the assessments of leisure-time physical activity with the season at blood sampling allowed for a good adjustment for sun exposure. Thereby, the difference seen in 25(OH)D concentrations was probably not caused by differences in vitamin D intake, milk intake or sun exposure.

This study has also some limitations. Misclassification in the frequency of consumption of sugar-sweetened beverages is possible with the use of a food frequency questionnaire. Intake of sugar-sweetened beverages over the previous year was an estimation given by each participant. However, differential bias is unlikely because answers inaccuracies can hardly be related to 25(OH)D concentrations and if there was misclassification, it could only lead to an underestimation of the effect. Also, it was difficult to state on the biological mechanism because the nutrients contained in colas that can be responsible for the lower 25(OH)D concentration (fructose, caffeine and phosphoric acid) were already present in several other foods. Despite introduction of season at blood sampling and leisure-time physical activity in the models, the adjustment for sun exposure was not perfect because the information about clothing style, sunscreen use and time spent outdoors other than leisure-time physical activity were not available. It is therefore possible that colas drinkers spent less time outdoors than non-drinkers and this could lead to residual confounding. Intake of colas may also be viewed as a proxy of an unhealthy lifestyle which could include higher BMI, waist-to-hip ratio, intake of alcohol and calorie and smoking habit, and lower intake of calcium and vitamin D and levels of physical activity. However, in our data, the correlations between such 25(OH)D determinants and 25(OH)D concentrations that were statistically significant remained so even after the introduction of total intake of colas in the model. Furthermore, this study population was limited to premenopausal women so inference to other populations is difficult to make. Finally, the cross-sectional design of this study does not allow causal interpretation.

## 5. Conclusions

In conclusion, we found that premenopausal women with higher intake of colas had lower circulating concentrations of 25(OH)D. To our knowledge, this is the first report of this association in such type of population. Considering the importance of vitamin D concentrations in the maintenance of a good health and the possible links of vitamin D deficiency to some diseases, our findings need further investigation. In our study population, the intake of colas was not very high, but in other populations this consumption can be more significant and may affect 25(OH)D concentrations in a greater manner. Considering the high consumption of this type of beverage in the general population, further studies are needed to elucidate the findings of the present study.

## References

[1] Nikpartow N., Danyliw A.D., Whiting S.J., Lim H.J., Vatanparast H. (2012). Beverage consumption patterns of canadian adults aged 19 to 65 years. Public Health Nutr..

[2] Lustig R.H., Schmidt L.A., Brindis C.D. (2012). Public health: The toxic truth about sugar. Nature.

[3] Merchant A.T., Tripathi A., Pervaiz F. (2010). Available energy from soft drinks: More than the sum of its parts. Public Health Nutr..

[4] Brown C.M., Dulloo A.G., Montani J.P. (2008). Sugary drinks in the pathogenesis of obesity and cardiovascular diseases. Int. J. Obes. (Lond.).

[5] Tucker K.L., Morita K., Qiao N., Hannan M.T., Cupples L.A., Kiel D.P. (2006). Colas, but not other carbonated beverages, are associated with low bone mineral density in older women: The framingham osteoporosis study. Am. J. Clin. Nutr..

[6] Mazariegos-Ramos E., Guerrero-Romero F., Rodriguez-Moran M., Lazcano-Burciaga G., Paniagua R., Amato D. (1995). Consumption of soft drinks with phosphoric acid as a risk factor for the development of hypocalcemia in children: A case-control study. J. Pediatr..

[7] Guerrero-Romero F., Rodriguez-Moran M., Reyes E. (1999). Consumption of soft drinks with phosphoric acid as a risk factor for the development of hypocalcemia in postmenopausal women. J. Clin. Epidemiol..

[8] Liu H., Heaney A.P. (2011). Refined fructose and cancer. Expert Opin. Ther. Targets.

[9] Michaud D.S., Fuchs C.S., Liu S., Willett W.C., Colditz G.A., Giovannucci E. (2005). Dietary glycemic load, carbohydrate, sugar, and colorectal cancer risk in men and women. Cancer Epidemiol. Biomarkers Prev..

[10] Friberg E., Wallin A., Wolk A. (2011). Sucrose, high-sugar foods, and risk of endometrial cancer—A population-based cohort study. Cancer Epidemiol. Biomarkers Prev..

[11] Tavani A., Giordano L., Gallus S., Talamini R., Franceschi S., Giacosa A., Montella M., La Vecchia C. (2006). Consumption of sweet foods and breast cancer risk in italy. Ann. Oncol..

[12] Ross A.C., Manson J.E., Abrams S.A., Aloia J.F., Brannon P.M., Clinton S.K., Durazo-Arvizu R.A., Gallagher J.C., Gallo R.L., Jones G. (2011). The 2011 report on dietary reference intakes for calcium and vitamin D from the institute of medicine: What clinicians need to know. J. Clin. Endocrinol. Metab..

[13] Mezza T., Muscogiuri G., Sorice G.P., Prioletta A., Salomone E., Pontecorvi A., Giaccari A. (2012). Vitamin D deficiency: A new risk factor for type 2 diabetes?. Ann. Nutr. Metab..

[14] Gagnon C., Lu Z.X., Magliano D.J., Dunstan D.W., Shaw J.E., Zimmet P.Z., Sikaris K., Ebeling P.R., Daly R.M. (2012). Low serum 25-hydroxyvitamin D is associated with increased risk of the development of the metabolic syndrome at five years: Results from a national, population-based prospective study (the australian diabetes, obesity and lifestyle study: Ausdiab). J. Clin. Endocrinol. Metab..

[15] Tamez H., Thadhani R.I. (2012). Vitamin D and hypertension: An update and review. Curr. Opin. Nephrol. Hypertens..

[16] Giovannucci E. (2005). The epidemiology of vitamin D and cancer incidence and mortality: A review (United States). Cancer Causes Control.

[17] Holick M.F. (2006). Vitamin D: Its role in cancer prevention and treatment. Prog. Biophys. Mol. Biol..

[18] Shao T., Klein P., Grossbard M.L. (2012). Vitamin D and breast cancer. Oncologist.

[19] Mohr S.B., Gorham E.D., Alcaraz J.E., Kane C.J., Macera C.A., Parsons J.K., Wingard D.L., Garland C.F. (2011). Serum 25-hydroxyvitamin D and prevention of breast cancer: Pooled analysis. Anticancer Res..

[20] Chen P., Hu P., Xie D., Qin Y., Wang F., Wang H. (2010). Meta-analysis of vitamin D,calcium and the prevention of breast cancer. Breast Cancer Res. Treat..

[21] Amir E., Cecchini R.S., Ganz P.A., Costantino J.P., Beddows S., Hood N., Goodwin P.J. (2012). 25-hydroxyvitamin D, obesity, and associated variables as predictors of breast cancer risk and tamoxifen benefit in NSABP-P1. Breast Cancer Res. Treat..

[22] Jones G. (2012). Metabolism and biomarkers of vitamin D. Scand. J. Clin. Lab. Invest. Suppl..

[23] Whiting S.J., Langlois K.A., Vatanparast H., Greene-Finestone L.S. (2011). The vitamin D status of canadians relative to the 2011 dietary reference intakes: An examination in children and adults with and without supplement use. Am. J. Clin. Nutr..

[24] Garcia-Contreras F., Paniagua R., Avila-Diaz M., Cabrera-Munoz L., Martinez-Muniz I., Foyo-Niembro E., Amato D. (2000). Cola beverage consumption induces bone mineralization reduction in ovariectomized rats. Arch. Med. Res..

[25] Olson M.L., Maalouf N.M., Oden J.D., White P.C., Hutchison M.R. (2012). Vitamin D deficiency in obese children and its relationship to glucose homeostasis. J. Clin. Endocrinol. Metab..

[26] Douard V., Asgerally A., Sabbagh Y., Sugiura S., Shapses S.A., Casirola D., Ferraris R.P. (2010). Dietary fructose inhibits intestinal calcium absorption and induces vitamin D insufficiency in CKD. J. Am. Soc. Nephrol..

[27] Portale A.A., Halloran B.P., Murphy M.M., Morris R.C. (1986). Oral intake of phosphorus can determine the serum concentration of 1,25-dihydroxyvitamin D by determining its production rate in humans. J. Clin. Investig..

[28] Calvo M.S. (1993). Dietary phosphorus, calcium metabolism and bone. J. Nutr..

[29] Diorio C., Pollak M., Byrne C., Masse B., Hebert-Croteau N., Yaffe M., Cote G., Berube S., Morin C., Brisson J. (2005). Insulin-like growth factor-I,IGF-binding protein-3, and mammographic breast density. Cancer Epidemiol. Biomarkers Prev..

[30] Brisson J., Berube S., Diorio C., Sinotte M., Pollak M., Masse B. (2007). Synchronized seasonal variations of mammographic breast density and plasma 25-hydroxyvitamin D. Cancer Epidemiol. Biomarkers Prev..

[31] Wolf A.M., Hunter D.J., Colditz G.A., Manson J.E., Stampfer M.J., Corsano K.A., Rosner B., Kriska A., Willett W.C. (1994). Reproducibility and validity of a self-administered physical activity questionnaire. Int. J. Epidemiol..

[32] Ainsworth B.E., Haskell W.L., Leon A.S., Jacobs D.R., Montoye H.J., Sallis J.F., Paffenbarger R.S. (1993). Compendium of physical activities. Med. Sci. Sports Exerc..

[33] Caan B.J., Slattery M.L., Potter J., Quesenberry C.P., Coates A.O., Schaffer D.M. (1998). Comparison of the block and the willett self-administered semiquantitative food frequency questionnaires with an interviewer-administered dietary history. Am. J. Epidemiol..

[34] Willett W.C., Sampson L., Stampfer M.J., Rosner B., Bain C., Witschi J., Hennekens C.H., Speizer F.E. (1985). Reproducibility and validity of a semiquantitative food frequency questionnaire. Am. J. Epidemiol..

[35] Sinotte M., Diorio C., Berube S., Pollak M., Brisson J. (2009). Genetic polymorphisms of the vitamin D binding protein and plasma concentrations of 25-hydroxyvitamin D in premenopausal women. Am. J. Clin. Nutr..

[36] Jaaskelainen T., Knekt P., Marniemi J., Sares-Jaske L., Mannisto S., Heliovaara M., Jarvinen R. (2012). Vitamin D status is associated with sociodemographic factors, lifestyle and metabolic health. Eur. J. Nutr..

[37] Beydoun M.A., Boueiz A., Shroff M.R., Beydoun H.A., Wang Y., Zonderman A.B. (2010). Associations among 25-hydroxyvitamin D, diet quality, and metabolic disturbance differ by adiposity in adults in the United States. J. Clin. Endocrinol. Metab..

[38] Smith S., Swain J., Brown E.M., Wyshak G., Albright T., Ravnikar V.A., Schiff I. (1989). A preliminary report of the short-term effect of carbonated beverage consumption on calcium metabolism in normal women. Arch. Intern. Med..

[39] Ventura E.E., Davis J.N., Goran M.I. (2011). Sugar content of popular sweetened beverages based on objective laboratory analysis: Focus on fructose content. Obesity (Silver Spring).

[40] Lee O., Bruce W.R., Dong Q., Bruce J., Mehta R., O’Brien P.J. (2009). Fructose and carbonyl metabolites as endogenous toxins. Chem. Biol. Interact..

[41] Stanhope K.L., Schwarz J.M., Keim N.L., Griffen S.C., Bremer A.A., Graham J.L., Hatcher B., Cox C.L., Dyachenko A., Zhang W. (2009). Consuming fructose-sweetened,not glucose-sweetened, beverages increases visceral adiposity and lipids and decreases insulin sensitivity in overweight/obese humans. J. Clin. Investig..

[42] Massey L.K., Whiting S.J. (1993). Caffeine, urinary calcium, calcium metabolism and bone. J. Nutr..

[43] Hernandez-Avila M., Stampfer M.J., Ravnikar V.A., Willett W.C., Schiff I., Francis M., Longcope C., McKinlay S.M., Longscope C. (1993). Caffeine and other predictors of bone density among pre- and perimenopausal women. Epidemiology.

[44] Amato D., Maravilla A., Montoya C., Gaja O., Revilla C., Guerra R., Paniagua R. (1998). Acute effects of soft drink intake on calcium and phosphate metabolism in immature and adult rats. Rev. Investig. Clin..

